# Comparison of COVID-19 epidemic among Czech dentists and the Czech general population

**DOI:** 10.1038/s41598-023-40427-8

**Published:** 2023-08-11

**Authors:** Jan Schmidt, Vojtech Perina, Jakub Suchanek, Jana Treglerova, Nela Pilbauerova, Ondrej Sanca, Jan Muzik, Roman Smucler

**Affiliations:** 1 Department of Dentistry, Charles University, Faculty of Medicine in Hradec Kralove and University Hospital Hradec Kralove, 500 05 Hradec Kralove, Czech Republic; 2 Department of Oral and Maxillofacial Surgery, Masaryk University-Faculty of Medicine and University Hospital Brno, 625 00 Brno, Czech Republic; 3 Institute of Health Information and Statistics of the Czech Republic, 128 00 Prague, Czech Republic; 4 Institute of Biostatistics and Analyses, Faculty of Medicine, Masaryk University, 625 00 Brno, Czech Republic; 5 Department of Stomatology, General Teaching Hospital, 1st Faculty of Medicine, Charles University, 121 08 Prague, Czech Republic

**Keywords:** Diseases, Health occupations

## Abstract

Dentists are one of the professional groups most at risk for COVID-19 infection. Enhanced protective measures in dentistry have been adopted worldwide; however, it is unclear to what extent they were sufficient. To assess whether the protective measures outweighed the high infection risk, we compared COVID-19-related data between Czech dentists and the general Czech population. The data was obtained through a survey study attended by 15.8% of Czech Dental Chamber members. Data of the general population were acquired from the Czech Ministry of Health database. By the end of May 2022, COVID-19 full vaccination and 1st booster dose rates among study participants were 85.8% and 70.1%, respectively, which is significantly higher (p < 0.0001) compared to the Czech general population aged over 24 years (74.9% and 49.4%, respectively). To the same date, PCR/Antigen test verified COVID-19 prevalence among participants was 41.7%, and 49.9% among the general population (p < 0.0001). Prevalence and reinfection rates among individuals who received the 1st booster were significantly lower than among individuals without the booster or full vaccination (p < 0.0001). Persons who received the booster showed a faster return to work, shorter and different types of complications. Willingness to future vaccination was positive among 79.7% of respondents. Mandatory vaccination for healthcare workers and the general population was supported by 62.0% and 49.0%, respectively. The results showed that the high risk of COVID-19 infection associated with dentistry did not lead to higher COVID-19 prevalence among respondents compared to the general population.

## Introduction

The COVID-19 pandemic, caused by SARS-CoV-2, has had a global impact on various professions, including dentistry. Dentists work in close proximity to patients while generating substantial amounts of aerosols. With a high patient turnover and limited space in dental offices, dentists face considerable occupational risks of COVID-19 infection, making them vulnerable to COVID-19 infection. In response, dentists have implemented increased protective measures, and many countries have prioritized their access to vaccination. These measures aim to mitigate the high risk of infection. However, its effectiveness remains uncertain.

Existing studies focusing solely on COVID-19-related data among dentists are inadequate for evaluating this situation, as they may be influenced by broader trends in the local population, not specific to dentistry. For instance, in a population where the pandemic’s impact was mild, lower morbidity rates can be expected even among dentists, and vice versa. Thus, it is crucial to compare COVID-19 data, such as prevalence or vaccination rates, among dentists with the corresponding data from the local general population. To the best of our knowledge, comprehensive studies with this setup are currently unavailable.

Despite being reported as one of the most affected countries by COVID-19, our previous study demonstrated that dental practices in the Czech Republic continued operating even during the most severe periods of the pandemic, with minimal restrictions on dental care provision^1^. Additionally, Czech dentists showed a high acceptance of vaccination^2^. These conditions provide a suitable setting to assess the effectiveness of protective measures and vaccination in mitigating the risk of infection associated with dentistry. However, current data regarding the COVID-19 prevalence among Czech dentists is unavailable. The latest available data from the first half of 2021 showed a low difference (1.7%) in COVID-19 prevalence between Czech dentists and the general population (without age disaggregation)^3^. It is important to acknowledge that this comparison was biased because dentists’ priority access to vaccination started in January 2021, while the general population did not have the same level of access until June 2021. Furthermore, the effectiveness of vaccination needs to be assessed with a sufficient time gap after its administration. To make a valid comparison between dentists and the adequately aged general population, it is necessary to consider a time when everyone had equal access to protective measures and a sufficient period had elapsed for these measures to take effect.

The aim of the study is to provide data on COVID-19 prevalence and vaccination among members of the Czech Dental Chamber and its timeline comparison with related data of the Czech general population from the beginning of the COVID-19 pandemic until the end of May 2022. Additionally, the study aims to describe the COVID-19 incapacity period and complications among respondents based on their vaccination status.

## Materials and methods

### Design

This study was designed by the Czech Dental Chamber experts in collaboration with dental practitioners and academic community members as a self-administered, cross-sectional survey. The study invitation with a link to the online questionnaire was sent to the officially registered e-mails of the Czech Dental Chamber members, together with information about the purpose of the study. The questionnaire was anonymous and did not contain any identifying personal information. Participation in the survey was voluntary, did not bring the respondents any remuneration or direct benefits, and was not protected against multiple entries. Information on survey aims, the anonymity of the questionnaire, and data protection were contents of the first section of the questionnaire. If the respondents did not confirm their consent to participate in the study and its terms and conditions, they were not allowed to continue filling out the questionnaire, nor was it possible to return the incomplete questionnaire. The full survey comprised a total of 67 items in 24 sections, although because of the adaptive nature of the questionnaire, not all respondents answered all items. The items were not randomized. The survey and the questionnaire content were approved and supervised by the Executive Board of the Czech Dental Chamber (Proceeding 04/2022, act 16.9) and approved by the Ethics Committee of The University Hospital Brno (reference number 01-120723/EK). The informed consent was waived by the Executive Board of the Czech Dental Chamber due to the retrospective nature of the study. The survey was performed in accordance with all relevant guidelines and regulations.

### Sample

A total of 10,193 members of the Czech Dental Chamber were addressed with an invitation to this survey via officially registered e-mail addresses. Study participation was possible from June 1, 2022, to July 1, 2022. The latest official report of the Czech Dental Chamber states that the number of its members was 11,147 as of December 31, 2021^4^. Thus, 91.4% of the members were addressed with this survey. The discrepancy between addressed members and the total number of members is due to the fact that not all members have officially registered their e-mail addresses. For dentists working in the Czech Republic, it is mandatory to be a member of the Czech Dental Chamber.

### Sample size relevancy

The minimum number of study participants was assessed as 372. Formula (1) was used for the calculation using the online Netquest calculator. (N) represents the study universe, i.e., Czech Dental Chamber members, (e) represents a margin of error set at 5%, (Z) represents a confidence level at 95%, and (p) represents a standard heterogeneity at 50%. The results of the study are statistically relevant as the minimal required number of participants was exceeded.

1 $$n =\frac{ N\cdot  {Z}^{2}\cdot  p\cdot  (1-p)}{(N-1)\cdot  {e}^{2}+{Z}^{2}\cdot  p\cdot  (1-p)}$$

Formula (1). Relevant sample size calculation.

### Data collection

The e-mail contained a link to an online questionnaire in Google Forms (Google, Mountain View, CA, USA). After completion, the data was stored in the Google Forms cloud database. All responses were provided within the set time period. Changing data after submission was not allowed. Data were downloaded after the end of the survey period.

### Analysis

The data were independently analyzed by three authors (Jan Schmidt, Vojtech Perina, and Jana Treglerova), with any discrepancies reviewed and resolved by the 4th author (Jakub Suchanek). Open-ended responses were analyzed and categorized based on their meaning, either by assigning them to a pre-defined answer or creating a new category when necessary. If a newly created category exceeded the threshold of five, it was included in the results; otherwise, the responses were categorized as “Others”. Blank responses were not included in the analysis.

For the purpose of the study, the period of infections was set as March 1, 2020, to May 31, 2022, while the time period of achieving full vaccinations was set as December 1, 2020, to May 31, 2022. The period of obtaining a booster dose was set as May 1, 2021, to May 31, 2022. Dates falling outside these ranges were considered invalid and excluded from the timeline analysis. Individuals who received one dose of the two-dose vaccine were considered partially vaccinated, while those who received one dose of a single-dose vaccine or two doses of a two-dose vaccine were considered fully vaccinated. The 1st and 2nd vaccine boosters were classified as the 1st and 2nd doses received after the full vaccination. The first case of infection is referred to as infection or primary infection, the second case as reinfection, and the third case as second reinfection. The prevalence rate represents the number of primary infections in the study population during the monitored period, while the reinfection rate represents the number of second infections in the study population during the same period.

COVID-19-related data of the general Czech population were obtained from a freely accessible Czech Ministry of Health database^5^. For comparison, only the data of individuals aged 24 years and older were utilized. The number of all individuals in this group was determined as individuals in the general Czech population older than 24 years, accordingly to the latest annual report of the Czech Statistical Office, i.e., as of December 31, 2021^6^.

The data were sorted and analyzed in Microsoft Office Excel (version 2106 for Windows, Microsoft Corporation, Redmond, WA, USA) and GraphPad Prism (version 8.0.0 for Windows, GraphPad Software, San Diego, CA, USA). The Chi-square test was used to evaluate dependency between the variables, and the Chi-square goodness of fit test was used to evaluate the fit between observed and expected frequencies. The confidence interval of the odds ratio was evaluated using Baptista–Pike and Woolf logit methods.

### Institutional review board statement

The survey and the content of the questionnaire were approved by the Ethics Committee of The University Hospital Brno and approved and supervised by the Executive Board of the Czech Dental Chamber.

## Results

### Response rate

A total of 1763 responses were received. The response rate was 17.3%. A total of 15.8% of all members of the Czech Dental Chamber participated in the survey. Out of them, 19 provided invalid information, e.g., infection or vaccination date out of the specified range, and were not included in the analyses. In total, 1744 respondents were included in the further analyses.

### Sex distribution

**Figure 1:**
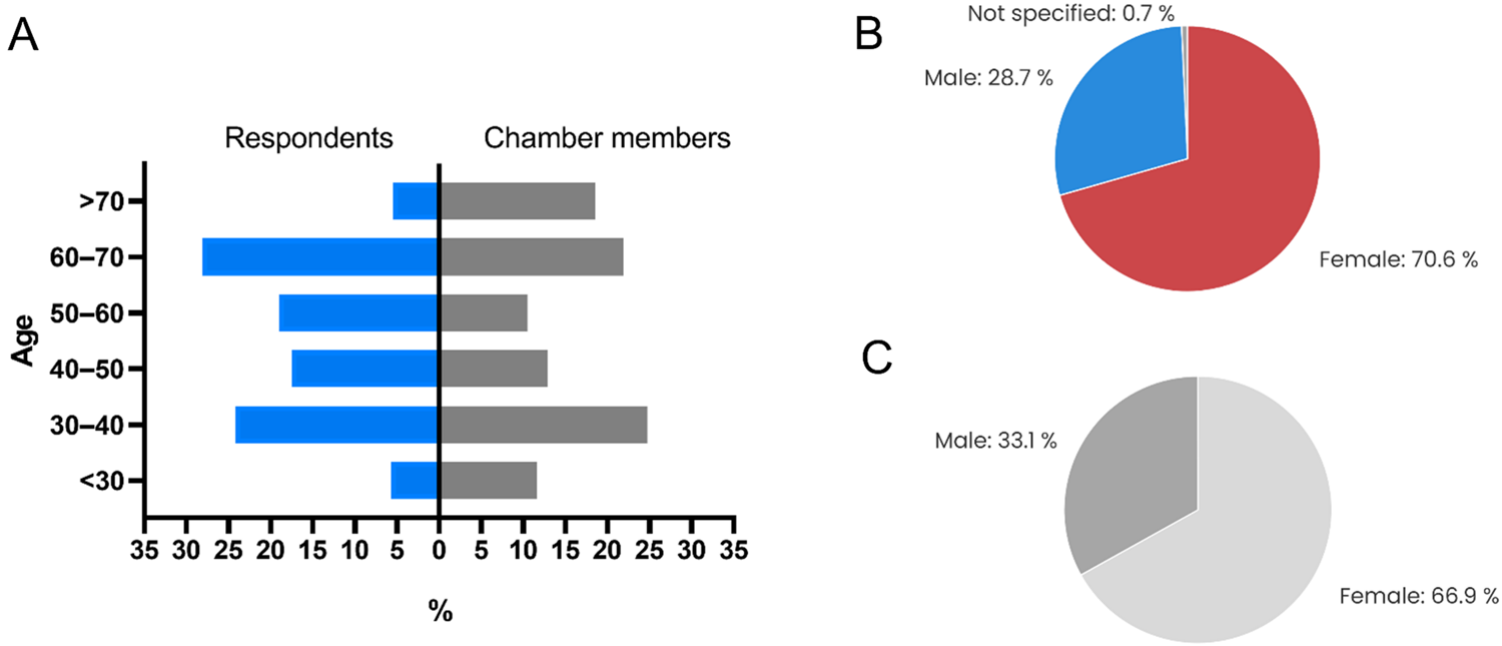
Age distribution (**A**) and sex distribution among respondents (**B**) and all Czech Dental Chamber members (**C**).

Information about sex was provided by 1731 respondents. Out of them, 1230 (70.6%) were females, 501 (28.7%) were males, and 13 (0.7%) did not specify their sex. Sex distributions among participants and Chamber members were distinct (p < 0.001; Chi-square goodness of fit test) and are presented in Fig. 1.

### Age distribution

Information about age was provided by 1738 respondents. Age distributions among participants and Chamber members were distinct (p < 0.0001; Chi-square goodness of fit test) and are presented in Fig. 1.

### Vaccination

Overall, 234 participants (13.4%) received no vaccine dose, 1510 (86.6%) participants received one vaccine dose, 1473 (84.5%) received two vaccine doses, 1208 (69.3%) received three vaccine doses, and 1 (0.1%) participant received four vaccine doses.

Out of all participants, 1496 (85.8%) were fully vaccinated, 1222 (70.1%) received 1st booster, and 3 (0.2%) received 2nd vaccine booster. Males were significantly more likely to get vaccinated than females (p = 0.007, OR = 1.55, 95% CI 1.13–2.1; Chi-square test and Baptista–Pike method). Age and sex distribution of vaccination are shown in Supplementary Fig. 1.

Of all 3678 vaccine doses administered, the vaccine types applied were: Comirnaty (Pfizer/BioNTech) 3290 (89.5%), Spikevax (Moderna) 243 (6.6%), Vaxzevria (AstraZeneca) 102 (2.8%), Janssen (Johnson & Johnson) 33 (0.9%), Nuvaxovid (Novavax) 3 (0.1%), and 7 (0.2%) did not know.

A total of 248 (14.2%) participants were classified as not fully vaccinated. The reason leading to avoiding full vaccination were: I do not want to be vaccinated (129, 52.0%), history of COVID-19 infection (37, 14.9%), laboratory tested high level of COVID-19 antibodies (26, 10.5%), medical contraindication of COVID-19 vaccination (12, 4.8%), I am waiting (9, 3.6%), insufficient time since receiving the 1st dose of vaccination (2, 0.8%), I do not want to answer/no answer (35, 14.1%).

### Protective measures

**Figure 2:**
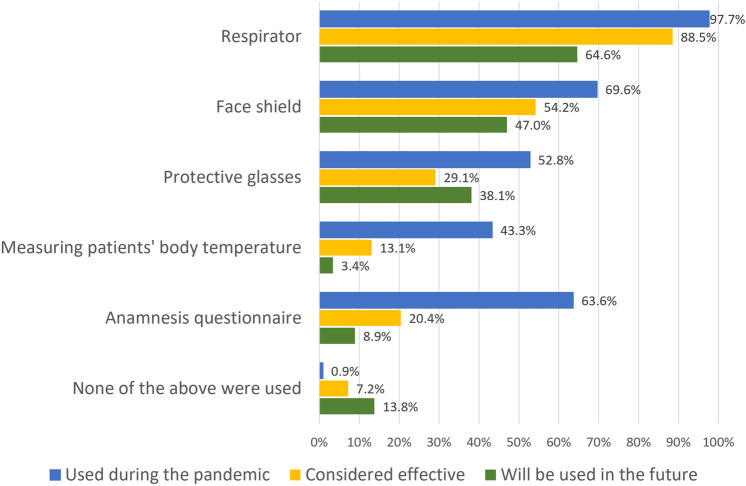
Protective measures used by respondents during the pandemic, their effectiveness assessment and future use.

Respondents were asked what protective measures they used during the pandemic and if they want to maintain them in the future. All 1744 respondents replied to this question. A total of 89% of respondents used eye barrier protection, such as protective glasses or a face shield. There was no statistically significant difference in COVID-19 prevalence based on a different approach to protective equipment. Results are presented in Fig. 2.

### COVID-19 infection

Overall, 845 (48.5%) respondents stated they were infected with COVID-19 between March 1, 2020, and May 31, 2022. COVID-19 prevalence sorted by sex revealed that the prevalence between men and women differed. Out of females, 621 (50.5%) replied they were infected with COVID-19, and 609 (49.5%) replied they were not. Among males, 220 (43.9%) stated they were infected with COVID-19, and 281 (56.1%) stated they were not. The difference is statistically significant (*p* = 0.013, OR = 0.77, 95% CI 0.625–0.94; Chi-square test and Baptista–Pike method). Details of prevalence based on age and sex are given in Supplementary Fig. 1. A total of 130 respondents admitted they were reinfected with COVID-19, which represents 7.5% of all respondents and 15.4% of respondents infected with COVID-19.

**Figure 3:**
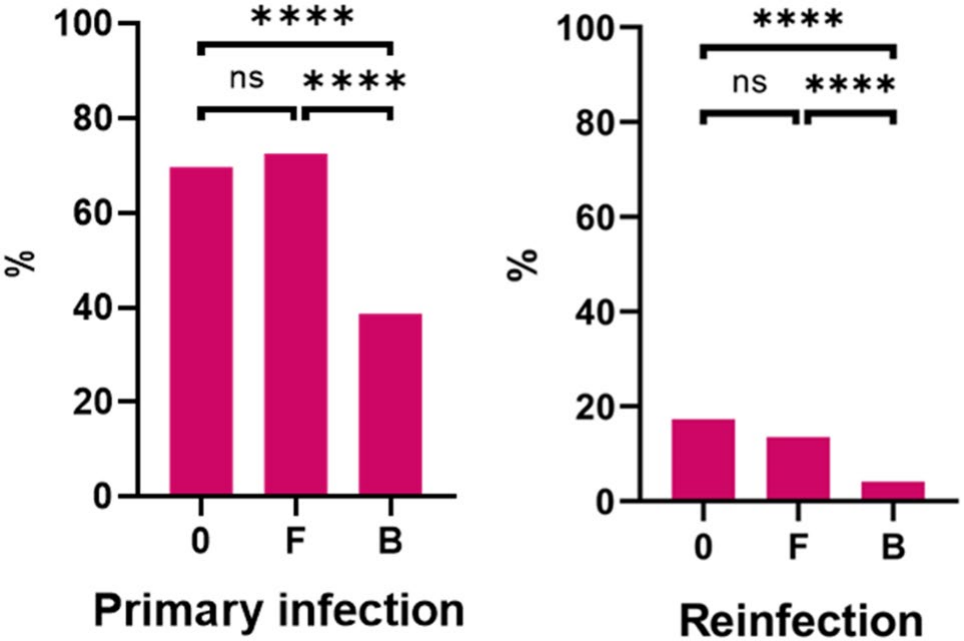
Primary infection and reinfection rates based on the vaccination status of respondents: not fully vaccinated (0), fully vaccinated without a 1st booster dose (F), fully vaccinated with a 1st booster dose (B). *ns* indicates statistical insignificance, **** indicate *p* < 0.0001, Chi-square test.

COVID-19 prevalence among 248 respondents who were not fully vaccinated was 69.8%. The COVID-19 prevalence rate among 274 respondents who achieved full vaccination but did not receive 1st booster dose was 72.6%. Compared to the group without full vaccination, the difference is not significant (*p* = 0.469; Chi-square test). Among 1222 respondents who received 1st booster vaccine dose, the COVID-19 prevalence rate was 38.7%. In comparison with the group of respondents who were not fully vaccinated, the difference is significant (*p* < 0.0001, OR = 0.27, 95% CI 0.20–0.37; Chi-square test and Baptista–Pike method). The difference between respondents with 1st booster vaccine dose and full vaccination is also significant (*p* < 0.0001, OR = 0.24, 95% CI = 0.18–0.32; Chi-square test and Baptista–Pike method) (Fig. 3).

The COVID-19 reinfection rate among 248 respondents who were not fully vaccinated was 17.3%. The COVID-19 prevalence rate among 274 respondents who achieved full vaccination but did not receive 1st booster dose was 13.5%. Compared to the group without full vaccination, the difference is not significant (*p* = 0.231, Chi-square test). Among 1222 respondents who received 1st booster vaccine dose, the COVID-19 reinfection rate was 4.1%. In comparison with the group of respondents who were not fully vaccinated, the difference is significant (*p* < 0.0001, OR = 0.20, 95% CI 0.13–0.31; Chi-square test and Baptista–Pike method). The difference between respondents with 1st booster vaccine dose and full vaccination is also significant (*p* < 0.0001, OR = 0.27, 95% CI 0.18–0.42; Chi-square test and Baptista–Pike method) (Fig. 3).

**Figure 4:**
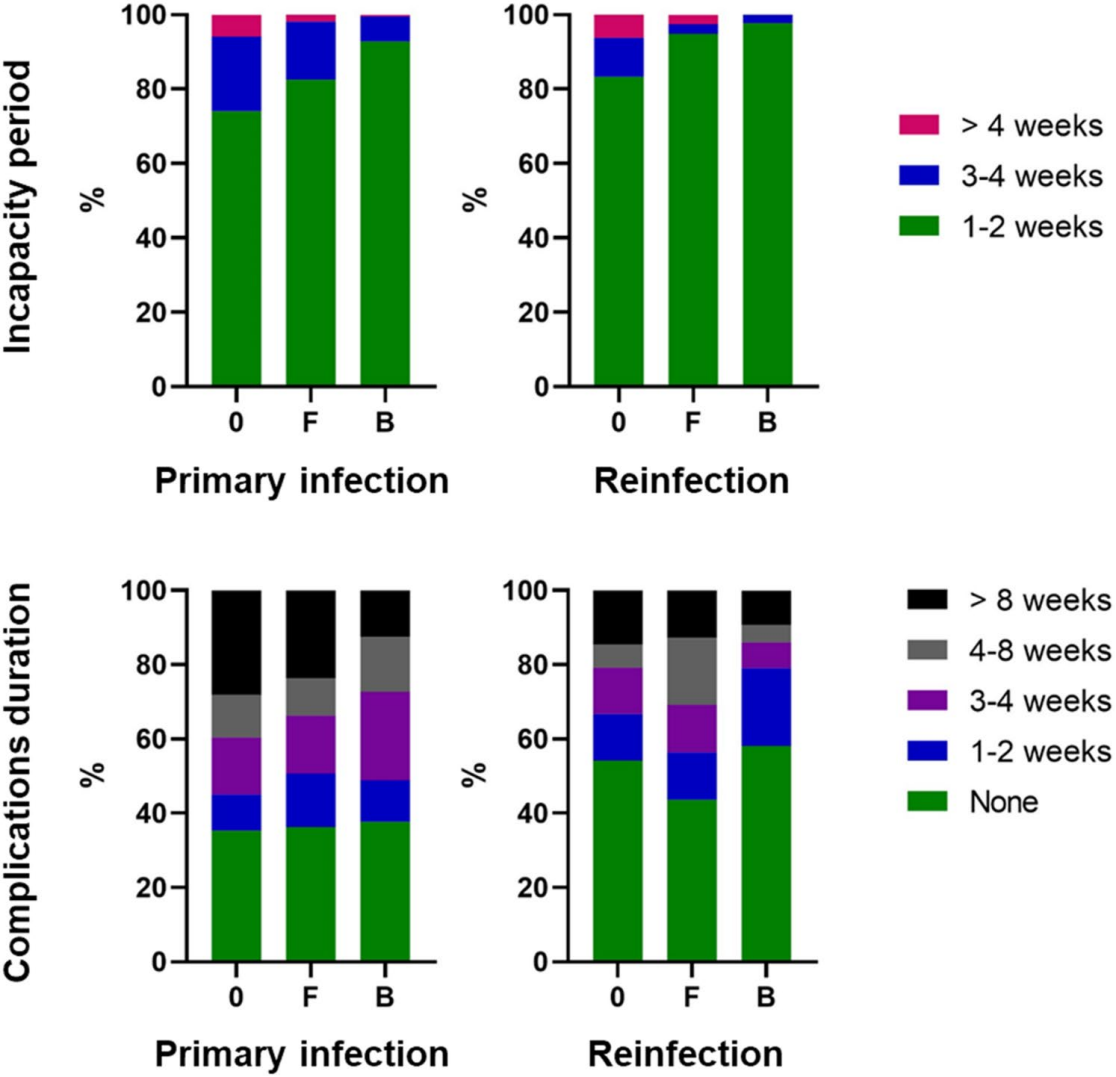
The incapacity period and duration of complications resulting from COVID-19 primary infection and reinfection among respondents sorted by their vaccination status at the time when they were infected: not fully vaccinated (0), fully vaccinated without a 1st booster dose (F), fully vaccinated with a 1st booster dose (B). Detailed data, including statistical analyses, are included in Supplementary Tables 1 and 2.

The incapacity period after COVID-19 infection, i.e., the time after which it was possible to return to work, was assessed for first and second infections. For the 845 cases of primary infection, the general incapacity period was as follows: 1–2 weeks 681 respondents (80.6%), 3–4 weeks 133 (15.7%), and more than 4 weeks 31 (3.7%). For the 130 cases of reinfection, the general incapacity period was as follows: 1–2 weeks 119 respondents (91.5%), 3–4 weeks 7 (5.4%), and more than 4 weeks 4 (3.1%). The long-term primary infection incapacity period, i.e., lasting longer than 4 weeks, was statistically significantly more frequent among persons without vaccination compared to persons with full vaccination or a booster (*p* = 0.043, OR = 3.25, 95% CI 1.05–10.31, and *p* = 0.0008, OR = 13.78, 95% CI 2.35–142.7, respectively; Chi-square test and Baptista–Pike method). The results are graphically displayed in Fig. 4. For a detailed statistical analysis of the length of the primary infection and reinfection incapacity period divided by vaccination status, see Supplementary Table 1.

Out of 845 infected respondents, 540 (63.9%) reported long-term complications, and 305 (36.1%) reported no long-term complications after infection. Of the 130 cases of reinfection, 61 (46.9%) were associated with long-term complications, and 69 (53.1%) were not. The frequency of complications lasting longer than 8 weeks after primary infection was higher among persons without full vaccination and with full vaccination compared to persons with a 1st booster dose (*p* < 0.0001, OR = 2.73, 95% CI 1.75–4.19, and *p* = 0.0042, OR = 2.17, 95% CI 1.29–3.765, respectively; Chi-square test and Baptista–Pike method). In the case of reinfections, the duration of complications differed based on the vaccination status; however, not significantly. The results are graphically displayed in Fig. 4. For a detailed statistical analysis of the length of the primary infection and reinfection long-term complications divided by vaccination status, see Supplementary Table 2.

Furthermore, differences between the types of complications, such as taste loss, smell loss, fatigue, respiratory difficulties, joint pain, headache, sleep difficulties, concentration difficulties, or hair loss, based on vaccination status, were analyzed. For instance, loss of smell and taste was significantly lowest among individuals with a 1st booster dose. The graphical display and statistical analysis of the types of complications based on the vaccination status are presented in Supplementary Fig. 3 and Supplementary Table 3.

Out of 845 respondents with a history of COVID-19 infection, 627 (74.2%) stated a source of COVID-19 primary infection. Out of 130 respondents reinfected with COVID-19, 91 (70.0%) stated a source of COVID-19 reinfection. The source of primary infection was stated as follows: Home 49.6%, Work 31.7% (Work—infected from a patient 11.8%, Work—the source of infection not specified 10.8%, Work—from an employee 9.1%), and Other 18.7% (N = 627). The number of infections in the home environment compared to the work environment is statistically significantly higher (p < 0.0001, OR = 2.44, 95% CI 1.90–3.14; Chi-square test and Baptista–Pike method). The source of reinfection was stated as follows: Home 39.6%, Work 42.9% (Work—infected from a patient 20.9%, Work—the source of infection not specified 14.3%, Work—from an employee 7.7%), Other 17.6% (N = 91). The number of reinfections in the home environment compared to the work environment is statistically insignificant (p = 0.624; Chi-square test).

Out of 845 infected respondents, six were hospitalized due to COVID-19 infection. The infection-hospitalization ratio was 0.7%. None of the hospitalized individuals was fully vaccinated. No reinfection led to hospitalization.

### Comparison with the Czech general population

**Figure 5:**
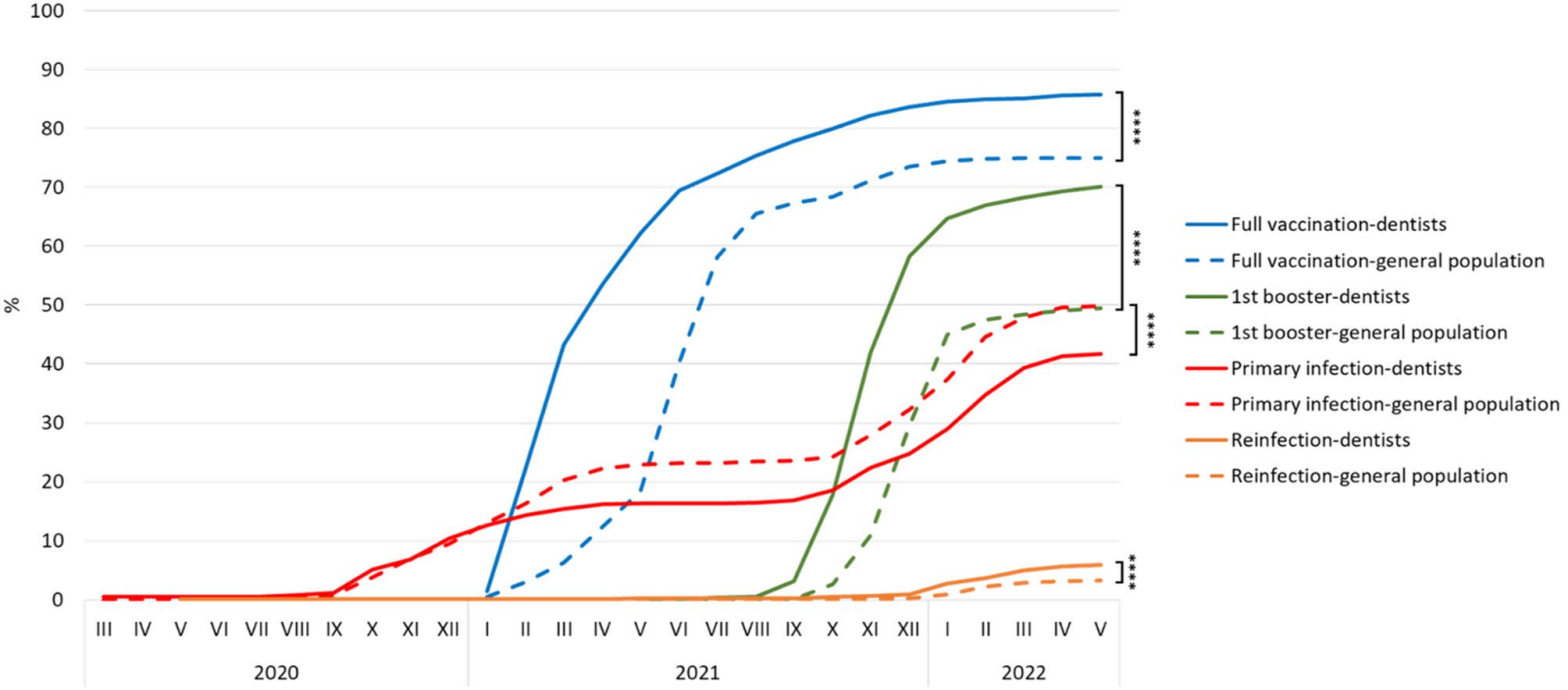
COVID-19 full vaccination, 1st booster vaccination, primary infection, and reinfection rates among respondents and the Czech general population, ****p < 0.0001, Chi-square test. Detailed timeline data are included in Supplementary Table 4.

The full vaccination rate among the respondents at the end of May 2022 was 85.8%. At the same time, the full vaccination rate among the Czech general population over 24 years was 74.9%. The difference is statistically significant (*p* < 0.0001, OR = 2.02, 95% CI 1.76–2.31; Chi-square test and Woolf method). 1st booster rate among the respondents at the end of May 2022 was 70.1%. At the same time, 1st booster rate among the Czech general population over 24 years was 49.4%. The difference is statistically significant (*p* < 0.0001, OR = 2.39, 95% CI 2.16–2.65; Chi-square test and Woolf method). The timeline comparison of full vaccination and 1st booster rates is demonstrated in Fig. 5.

The COVID-19 PCR/Ag-verified primary infection rate among the respondents at the end of May 2022 was 41.7%. At the same time, the PCR/Ag-verified primary infection rate among the Czech general population older than 24 years was 49.9%. The difference is statistically significant (*p* < 0.0001, OR = 0.72, 95% CI 0.65–0.79; Chi-square test and Woolf method).

The COVID-19 PCR/Ag-verified reinfection rate among the respondents at the end of May 2022 was 6.0%. At the same time, the PCR/Ag-verified reinfection rate among the Czech general population older than 24 years was 3.3%. The difference is statistically significant (*p* < 0.0001, OR = 1.88, 95% CI 1.54–2.29; Chi-square test and Woolf method). The timeline comparison of primary infection and reinfection rates is demonstrated in Fig. 5.

### Attitudes to mandatory and future COVID-19 vaccination

Respondents were asked about their approach to future vaccination and their opinion on mandatory vaccination of healthcare workers and the general population. All 1744 respondents replied to this question. Attitudes to future vaccination against COVID-19 were stated as follows: Yes 15.9%, Yes, only in case of an ongoing pandemic 11.2%, Yes, if the vaccine will be updated 24.0%, Yes, if necessary for work/traveling 28.6%, No 20.4%. Attitudes to mandatory vaccination against COVID-19 for healthcare professionals were stated as follows: Strongly agree 32.9%, Somewhat agree 29.1%, Neither agree nor disagree 4.2%, Somewhat disagree 13.7%, Strongly disagree 20.1%. Attitudes to mandatory vaccination against COVID-19 for the general population were stated as follows: Strongly agree 14.7%, Somewhat agree 34.3%, Neither agree nor disagree 6.7%, Somewhat disagree 20.0%, Strongly disagree 24.3%.

### COVID-19 impact on dental practices

Study participants were asked how the COVID-19 pandemic affected the number of patients, economic situation, and operativeness of their dental practices. All 1744 respondents provided answers to these questions. An increased number of patients was reported by 6.7% of respondents, no impact was reported by 38.5% of respondents, a decrease in patients was reported by 45.8% of respondents, and 9.1% of them did not know. The economic impact of the pandemic was positive for 4% of respondents, none for 27.4% of respondents, negative for 53.0% of respondents, and 15.5% did not know. The impact on professional operativeness among respondents was stated as follows: No impact 32.2%, One-time closure shorter than 1 week 23.8%, One-time closure longer than 1 week 30.3%, Repeated closure during the pandemic 8.2%, Permanent reduction in working hours 4.9%, and Permanent closure 0.5%.

## Discussion

At the end of May 2022, the reported COVID-19 prevalence rate among Czech dentists was 48.5%, and the reinfection rate was 7.5%. There was no statistically significant difference in prevalence rate or reinfection rate between persons with and without full vaccination against COVID-19. However, among persons who received the 1st booster vaccine dose, the prevalence and reinfection rates were statistically significantly lower. These results show that the 1st vaccine booster provided significant protection to dentists during the entire study period, but simple full vaccination without a booster did not. These conclusions are consistent with the recommendations of vaccine manufacturers, which state that revaccination is necessary to maintain adequate protection. Willingness to vaccinate in the future was unconditionally positive among 15.9% of respondents; among 63.8%, it was conditionally positive, and 20.4% of them had a negative attitude. Mandatory vaccination for healthcare workers and the general population is supported by 62.0% and 49.0%, respectively.

Compliance with vaccination against COVID-19 was also associated with a shorter incapacity period resulting from COVID-19 infection. Infected persons who were vaccinated had a significantly shorter incapacity period and faster return to work. Persons whose infection occurred after a booster dose of the vaccine showed the fastest return to work, followed by persons who became ill after achieving full vaccination but without a booster dose. It took the longest time to return to work for persons who became ill at a time when they were not vaccinated. The same applies to reinfections.

Significant differences depending on vaccination status were also observed in the duration of complications after COVID-19 infection. The duration of complications was shorter in vaccinated persons. Complications lasting longer than 8 weeks were significantly less frequent among subjects infected after receiving a booster dose compared to unvaccinated and fully vaccinated individuals without a booster dose. A similar trend was evident in the duration of complications after reinfections. Vaccination also had a notable effect on the type of complications that developed in respondents. Additionally, persons infected after receiving the 1st booster dose showed significantly fewer complications, such as loss of taste or smell, than those infected without vaccination or after full vaccination but without a booster dose. The booster also provided significant protection against hair loss compared to non-vaccinated subjects. On the other hand, people experiencing COVID-19 infection after receiving a booster dose more often had complications such as fatigue, joint pain, or headache. In summary, vaccination compliance led to reduced COVID-19 prevalence and reinfection rates, shorter complications, and a faster return to work after COVID-19 infection.

Czech dentists also showed high compliance with protective measures. Almost all respondents used respirators at work and rated them as effective. Most of them wanted to keep using respirators even after the end of the pandemic. A total of 89% of respondents used eye barrier protection, such as protective glasses or a face shield. On the other hand, the anamnestic questionnaires used by 63.6% of respondents were evaluated as effective by only one-fifth of respondents, and less than one-tenth wanted to maintain their use in the future. High compliance with work-related protective measures corresponds with the infection source analysis. The number of infections in the work environment compared to the home environment was statistically significantly lower. Interestingly, no significant differences in prevalence were found depending on the application of protective measures.

In order to evaluate how effectively Czech dentists resisted the infection of COVID-19, it is necessary to compare the results of this study with the data of the general Czech population. Full vaccination was achieved by 85.8% of respondents, while among the general Czech population over the age of 24, it was 74.9%. In the case of the 1st booster dose, it was 70.1% and 49.4%, respectively. The differences are statistically significant. The methodology of the Czech Ministry of Health did not allow verification of COVID-19 infection based only on symptoms but had to be based on an antigen or PCR test^7^. Therefore, it is necessary to apply the same methodology to the data of this study to compare prevalence and reinfection rates. PCR/Ag-verified COVID-19 prevalence among respondents was 41.7%, and among the general Czech population over 24 years of age, it was 49.9%. The prevalence among respondents is statistically significantly lower than among counterparts from the general Czech population. On the other hand, the PCR/Ag-verified reinfection rate was statistically significantly higher among study participants (6.0%) than among the Czech general population counterparts (3.3%). Among these reinfected study participants, the booster rate was remarkably low. Only 35.6% of them received a booster vaccine dose. In summary, revaccination was a decisive factor in protecting against COVID-19. This conclusion is particularly important as 49.0% of respondents do not want to receive an additional vaccine dose or would accept it only if necessary for work or travel.

The data also demonstrate the effect of healthcare workers’ priority access to vaccination against COVID-19 in the first half of 2021. While by January 2021, the prevalence of COVID-19 was similar between respondents and the general population, rates began to differ after the start of selective vaccination. After the vaccination became generally available, the prevalence curves followed the same trends.

A negative economic impact of the pandemic on dental practices was reported by 53% of respondents, and 45.8% of respondents stated it also led to a reduction in the number of patients. During the pandemic, 4.9% of respondents decided to reduce working hours permanently, and 0.5% of respondents closed their practice permanently. However, for most respondents, the pandemic led to a work restriction of no longer than 1 week without impacting routine operability. This indicates that the availability of dental care was minimally restricted during the pandemic.

A limitation of the study is that the data is based on a self-reported survey. However, another way of obtaining COVID-19-related data on dentists is not possible, as the official COVID-19 prevalence and vaccination databases are not sorted by profession. Another limitation of this study may be not reflecting on different subtypes of the SARS-CoV-2 virus. Individual subtypes showed different characteristics, e.g., rate of infectivity or complications. However, this limitation is due to the generally low rate of detection of virus variants in the Czech Republic, given the limited capacity of testing facilities. Variants of the virus were investigated only in a limited number of samples, mainly for the purposes of estimating the overall dynamics of the epidemic. It is likely that variants of the virus influenced the development of the epidemic among Czech dentists, but a valid conclusion would require data that are not available.

Comparison with studies by other authors is limited by different methodologies, external trends, and different time widows of the studies. Various studies are reporting on dentists' attitudes to vaccination against COVID-19, the psychological effect of COVID-19, or fears of infection. However, studies providing data on real vaccination and prevalence rates are scarce. In May 2022, Madathil et al. published a study reporting on the incidence of COVID-19 among 644 dentists across Canada^8^. Data for this study were collected via a self-reported online questionnaire from July 29, 2020, to February 12, 2021. It is one of the few studies comparing results with the local population, in this case, the general Canadian population. The incidence was 1084 per 100,000 dentists and 1864 per 100,000 people among the Canadian population. The authors conducted that uptake of vaccinations could be a possible explanation for the low infection rates of dentists, but the proportion of participants who had received at least one dose of the COVID-19 vaccine was low (≈ 5%) and cannot explain the low infection rates. In May 2021, Ferreira et al. reported on the morbidity of COVID-19 among Brazilian dentists From January to October 2020^9^. The cumulative incidence of COVID-19 among dentists was 18.70/1000; among the general Brazilian population, it was 17.71/1000, i.e., ≈ 5% lower. In April 2022, Tanaka et al. published a study reporting on COVID-19 spread and measures in dental and orofacial clinics in Japan^10^. The study was conducted in February 2021 via online questionnaires and included 51 workplaces. However, it did not describe the prevalence of medical staff but focused on infection control measures, hospital operability, contact with patients, and infection transmission. 92.2% of workplaces reported no staff members who were positive for COVID-19. The authors concluded that COVID-19 clusters are unlikely to occur in dental and oral surgical care settings if appropriate protective measures are implemented. The literature search shows that COVID-19-related studies among dentists are scant and current data are not available.

## Conclusions

Czech dentists demonstrated high compliance with the use of personal protective equipment and vaccination against COVID-19. Compared to the Czech general population, dentists had significantly higher rates of full vaccination and 1st booster doses and a lower prevalence of COVID-19. These findings suggest that the implementation of protective measures, particularly vaccination, has proven effective in mitigating the heightened risk of COVID-19 infection associated with the dental profession. Furthermore, the results indicate that the vaccinated individuals experienced a shorter incapacity period and encountered fewer complications following COVID-19 infection.

### Supplementary Information

Supplementary Information 1.

Supplementary Information 2.

## Data Availability

The datasets generated during and/or analysed during the current study are available from the corresponding author on reasonable request.
