# Supplementary material for: Comparison of COVID-19 epidemic among Czech dentists and the Czech general population
Source: Sci Rep. 2023 Aug 11;13:13104. doi: 10.1038/s41598-023-40427-8 (PMC10421873; doi:10.1038/s41598-023-40427-8)
Supplement: Supplementary file 1 — Supplementary Information 1. [file 41598_2023_40427_MOESM1_ESM.pdf]

What is your COVID-19 vaccination status?
What was the indication for vaccination?
Date of application of the 1st dose:
What type of vaccine was used?
Did you receive the 2nd dose of the vaccine?
Date of application of the 2nd dose:
What type of vaccine was used?
Did you receive the 3rd dose of the vaccine (booster)?
Date of application of the 3rd dose:
What type of vaccine was used?
Did you receive the 4th dose of the vaccine (booster)?
Application date of the 4th dose:
What type of vaccine was used?
For what reasons are you not fully vaccinated?

First COVID-19 infection

Were you infected with COVID in the period 1/3/2020 - 31/5/2022?
How was the COVID-19 infection diagnosed?
Where the treatment took place?
When did the disease start?
After how long was it possible to return to work?
For what reason was an earlier return to work not possible?
Did you suffer from any long-term complications after the illness? If yes, specify them.
How long did these problems last?
Do you know the infection source?
What was the source of the infection?

Second COVID-19 infection

In the period 1/3/2020 - 31/5/2022, did you suffer from the COVID disease for the second time?
How was the COVID-19 infection diagnosed?
Where the treatment took place?
When did the disease start?
After how long was it possible to return to work?
For what reason was an earlier return to work not possible?
Did you suffer from any long-term complications after the illness? If yes, specify them.
How long did these problems last?
Do you know the infection source?
What was the source of the infection?

Third COVID-19 infection

In the period 1/3/2020 - 31/5/2022, did you suffer from the COVID disease for the third time?
How was the COVID-19 infection diagnosed?
Where the treatment took place?
When did the disease start?
After how long was it possible to return to work?
For what reason was an earlier return to work not possible?
Did you suffer from any long-term complications after the illness? If yes, specify them.
How long did these problems last?
Do you know the infection source?
What was the source of the infection?

Have you experienced the disease of COVID-19 more than 3 times?
Was another work team member sick of COVID-19?
Who else got sick?
Did more than one employee get sick or one repeatedly?
What protective measures did you use during the epidemic?
What protective measures do you consider effective?

What protection do you consider unnecessary?
Will you use any protective measures in the future? Which?
What measures should the recommendation be extended to?
What additional measures were applied in your practice?
Do you agree with the mandatory vaccination of the general population?
Do you agree with the mandatory vaccination of healthcare workers?
What is your attitude to receiving additional COVID-19 vaccine dose?
How have COVID-19 and epidemic-related measures affected the number of your patients?
How have COVID-19 and measures related to the epidemic economically affected your dental practice?
Has COVID-19 and related measures affected the operation of your dental practice?
Sex
Age
Your Czech Dental Chamber regional branch:
Indicate your working status as of 31/5/2022:
Before the start of the COVID-19 epidemic, I worked:
You can add your comment here:
