# Supplementary material for: Comparison of COVID-19 epidemic among Czech dentists and the Czech general population
Source: Sci Rep. 2023 Aug 11;13:13104. doi: 10.1038/s41598-023-40427-8 (PMC10421873; doi:10.1038/s41598-023-40427-8)
Supplement: Supplementary file 2 — Supplementary Information 2. [file 41598_2023_40427_MOESM2_ESM.pdf]

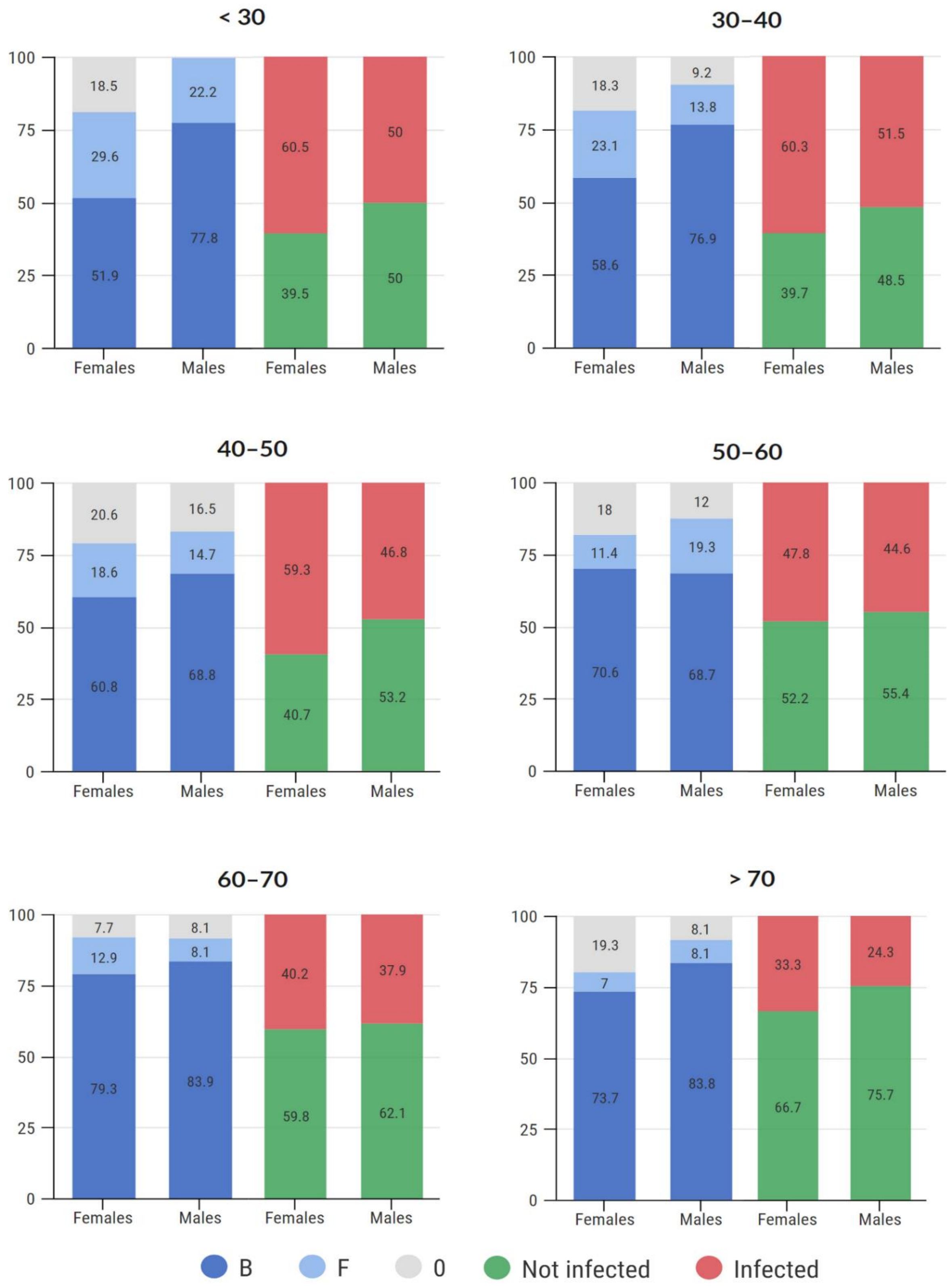

Supplementary Fig. 1. COVID-19 vaccination and prevalence based on respondents' age and sex. Vaccination data are sorted as follows: fully vaccinated with a 1st booster dose (B), fully vaccinated without a 1st booster dose (F), not fully vaccinated (0).

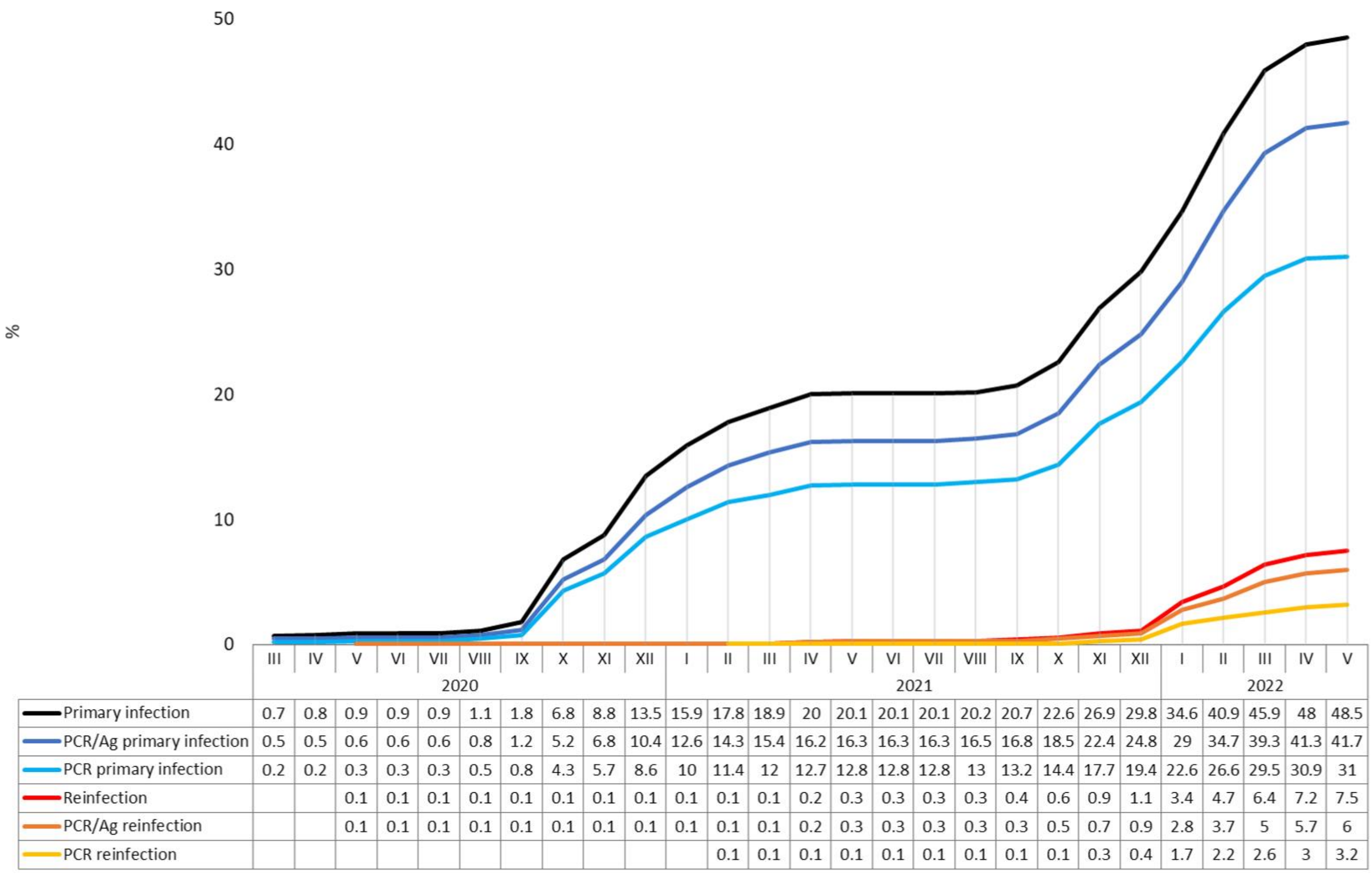

	III	IV	V	VI	VII	VIII	IX	X	XI	XII	I	II	III	IV	V	VI	VII	VIII	IX	X	XI	XII	I	II	III	IV	V
	2020										2021												2022				
Primary infection	0.7	0.8	0.9	0.9	0.9	1.1	1.8	6.8	8.8	13.5	15.9	17.8	18.9	20	20.1	20.1	20.1	20.2	20.7	22.6	26.9	29.8	34.6	40.9	45.9	48	48.5
PCR/Ag primary infection	0.5	0.5	0.6	0.6	0.6	0.8	1.2	5.2	6.8	10.4	12.6	14.3	15.4	16.2	16.3	16.3	16.3	16.5	16.8	18.5	22.4	24.8	29	34.7	39.3	41.3	41.7
PCR primary infection	0.2	0.2	0.3	0.3	0.3	0.5	0.8	4.3	5.7	8.6	10	11.4	12	12.7	12.8	12.8	12.8	13	13.2	14.4	17.7	19.4	22.6	26.6	29.5	30.9	31
Reinfection			0.1	0.1	0.1	0.1	0.1	0.1	0.1	0.1	0.1	0.1	0.1	0.2	0.3	0.3	0.3	0.3	0.4	0.6	0.9	1.1	3.4	4.7	6.4	7.2	7.5
PCR/Ag reinfection			0.1	0.1	0.1	0.1	0.1	0.1	0.1	0.1	0.1	0.1	0.1	0.2	0.3	0.3	0.3	0.3	0.3	0.5	0.7	0.9	2.8	3.7	5	5.7	6
PCR reinfection												0.1	0.1	0.1	0.1	0.1	0.1	0.1	0.1	0.1	0.3	0.4	1.7	2.2	2.6	3	3.2

Supplementary Fig. 2. Primary infection and reinfection rates among respondents from March 2020 to May 2022 based on the diagnostic method used. Primary infection and Reinfection lines represent data regardless of diagnostic method, including diagnosis based on clinical symptoms only.

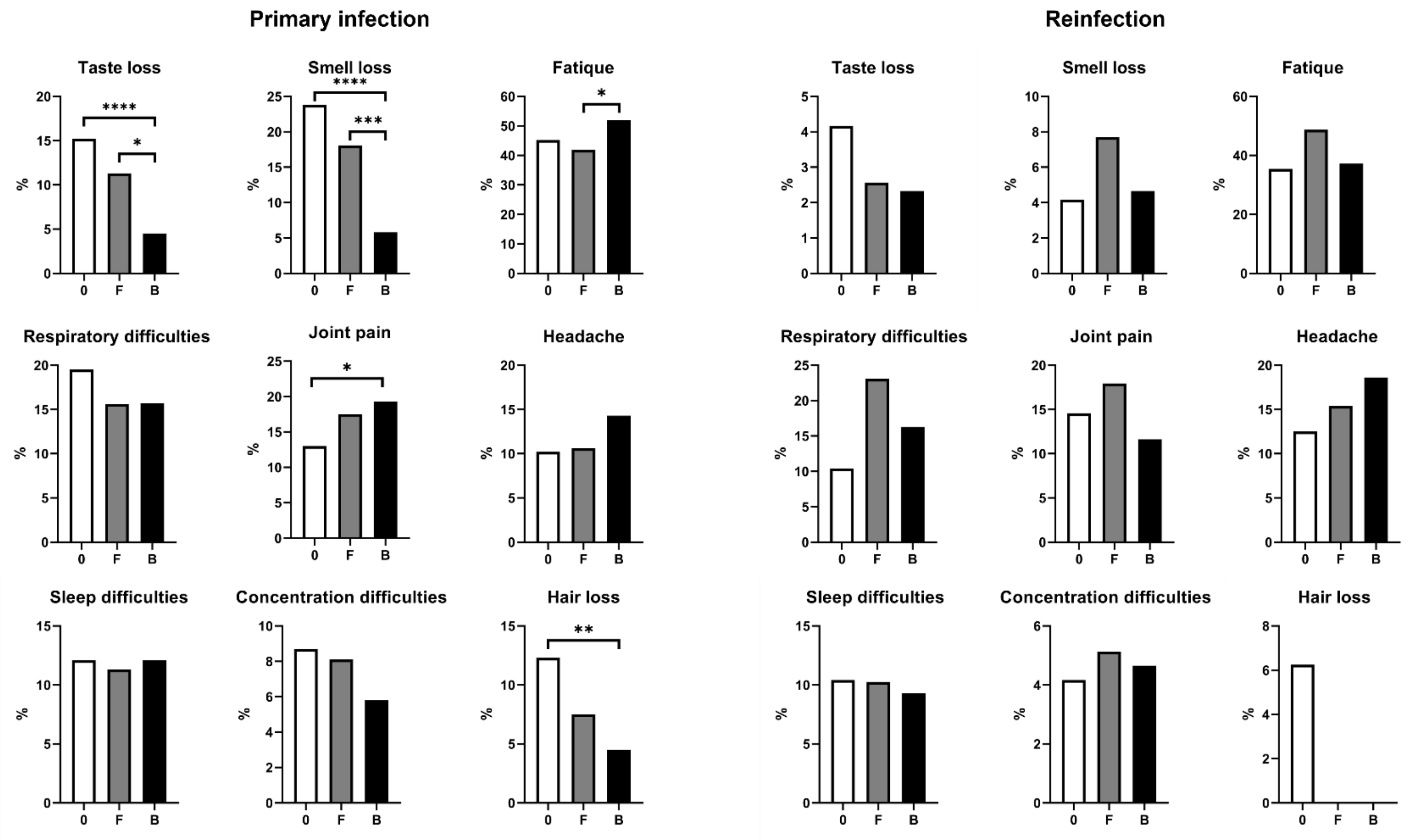

Supplementary Fig. 3. Type of complications resulting from COVID-19 primary infection and reinfection among respondents sorted by their vaccination status at the time when they were infected: not fully vaccinated (0), fully vaccinated without a 1st booster dose (F), fully vaccinated with a 1st booster dose (B); * $p \leq 0.05$, ** $p < 0.005$, *** $p < 0.001$, **** $p < 0.0001$. If not stated differently, differences are statistically insignificant. For primary infections, the chi-square test and Baptista-Pike method were used; for reinfections, Fisher's exact test and Baptista-Pike method were used. Detailed data, including statistical analyses, are included in Supplementary Table 3.

Supplementary Table 1. Incapacity periods resulting from COVID-19 primary infection and reinfection among respondents sorted by their vaccination status at the time when they were infected: not fully vaccinated (0), fully vaccinated without a 1st booster dose (F), fully vaccinated with a 1st booster dose (B). $p \leq 0.05$ are in bold. For primary infections, the chi-square test and Baptista-Pike method were used; for reinfections, Fisher's exact test and Baptista-Pike method were used.

**Primary infection**	**0**		**F**		**B**		**0 vs. F**			**0 vs. B**			**F vs. B**		
	**n**	**%**	**n**	**%**	**n**	**%**	**p**	**OR**	**95% CI**	**p**	**OR**	**95% CI**	**p**	**OR**	**95% CI**
**> 4 weeks**	27	5.8	3	1.9	1	0.4	**0.0434**	3.248	1.052-10.31	**0.0008**	13.78	2.347-142.7	0.1756	4.242	0.6258-55.32
**3-4 weeks**	93	20.1	25	15.6	15	6.7	0.2104	1.361	0.847-2.187	**<0.0001**	3.495	1.968-6.147	**0.005**	2.568	1.294-4.926
**1-2 weeks**	342	74.0	132	82.5	207	92.8	**0.03**	0.6045	0.384-0.950	**<0.0001**	0.2203	0.0636-0.3761	**0.0018**	0.3644	0.1852-0.6872

**Reinfection**	**0**		**F**		**B**		**0 vs. F**			**0 vs. B**			**F vs. B**		
	**n**	**%**	**n**	**%**	**n**	**%**	**p**	**OR**	**95% CI**	**p**	**OR**	**95% CI**	**p**	**OR**	**95% CI**
**> 4 weeks**	3	6.3	1	2.6	0	0	0.6245	2.533	0.362-33.66	0.244	∞	0.7906-∞	0.4756	∞	0.1225-∞
**3-4 weeks**	5	10.0	1	2.6	1	2.3	0.2177	4.419	0.547-53.26	0.2073	4.884	0.6076-58.73	>0.9999	1.105	0.0568-21.45
**1-2 weeks**	40	83.0	37	95.0	42	98.0	0.1744	0.2703	0.056-1.261	**0.0324**	0.119	0.0151-0.8742	0.6023	0.4405	0.0298-3.944

Supplementary Table 2. Duration of complications resulting from COVID-19 primary infection and reinfection among respondents sorted by their vaccination status at the time when they were infected: not fully vaccinated (0), fully vaccinated without a 1st booster dose (F), fully vaccinated with a 1st booster dose (B). $p \leq 0.05$ are in bold. For primary infections, the chi-square test and Baptista-Pike method were used; for reinfections, Fisher's exact test and Baptista-Pike method were used.

**Primary infection**	**0**		**F**		**B**		**0 vs. F**			**0 vs. B**			**F vs. B**		
	**n**	**%**	**n**	**%**	**n**	**%**	**p**	**OR**	**95% CI**	**p**	**OR**	**95% CI**	**p**	**OR**	**95% CI**
**> 8 weeks**	130	28.1	38	23.8	28	12.6	0.2813	1.257	0.837-1.897	**<0.0001**	2.727	1.748-4.193	**0.0042**	2.169	1.290-3.765
**4-8 weeks**	53	11.5	16	10.0	33	14.8	0.5109	1.166	0.660-2.079	0.2183	0.746	0.467-1.209	0.1656	0.6397	0.334-1.212
**3-4 weeks**	71	15.4	25	15.6	53	23.8	0.9382	0.981	0.592-1.609	**0.0075**	0.582	0.395-0.8720	0.0510	0.594	0.356-0.9937
**1-2 weeks**	45	9.7	23	14.4	25	11.2	0.1054	0.643	0.376-1.096	0.5516	0.855	0.519-1.452	0.3563	1.330	0.735-2.375
**None**	163	35.3	58	36.3	84	37.7	0.8254	0.959	0.659-1.398	0.5421	0.902	0.652-1.260	0.7769	0.9409	0.617-1.420

**Reinfection**	**0**		**F**		**B**		**0 vs. F**			**0 vs. B**			**F vs. B**		
	**n**	**%**	**n**	**%**	**n**	**%**	**p**	**OR**	**95% CI**	**p**	**OR**	**95% CI**	**p**	**OR**	**95% CI**
**> 8 weeks**	7	14.6	5	12.8	4	9.3	>0.9999	1.161	0.352-3.605	0.5298	1.665	0.465-5.374	0.7298	1.434	0.380-4.955
**4-8 weeks**	3	6.3	7	17.9	2	4.7	0.1045	0.305	0.008-1.263	>0.9999	1.367	0.267-7.973	0.0784	4.484	0.861-22.14
**3-4 weeks**	6	12.5	5	12.8	3	7.0	>0.9999	0.971	0.270-3.155	0.4914	1.905	0.506-7.274	0.4684	1.961	0.459-7.772
**1-2 weeks**	6	12.5	5	12.8	9	20.9	>0.9999	0.971	0.270-3.155	0.3971	0.540	0.167-1.692	0.3888	0.556	0.189-1.908
**None**	26	54.2	17	43.6	25	58.1	0.3909	1.529	0.663-3.660	0.8328	0.851	0.374-1.902	0.2689	0.556	0.245-1.345

Supplementary Table 3. Type of complications resulting from COVID-19 primary infection and reinfection among respondents sorted by their vaccination status at the time when they were infected: not fully vaccinated (0), fully vaccinated without a 1st booster dose (F), fully vaccinated with a 1st booster dose (B). $p \leq 0.05$ are in bold. For primary infections, the chi-square test and Baptista-Pike method were used; for reinfections, Fisher's exact test and Baptista-Pike method were used.

**Primary infection**	**0**		**F**		**B**		**0 vs. F**			**0 vs. B**			**F vs. B**		
	**n**	**%**	**n**	**%**	**n**	**%**	**p**	**OR**	**95% CI**	**p**	**OR**	**95% CI**	**p**	**OR**	**95% CI**
Taste loss	70	15.2	18	11.3	10	4.5	0.2223	1.409	0.8271-2.447	**<0.0001**	3.804	1.924-7.862	**0.0121**	2700	1.224-5.802
Smell loss	110	23.8	29	18.1	13	5.8	0.1369	0.6645	0.8998-2.202	**<0.0001**	5.048	2.824-8.981	**0.0001**	3576	1.802-7.347
Fatigue	209	45.2	67	41.9	116	52.0	0.4605	0.6645	0.8022-1.658	0.0959	0.762	0.5520-1.049	0.05	0.6645	0.4465-1.002
Respiratory difficulties	90	19.5	25	15.6	35	15.7	1.083	1.306	0.8105-2.104	1.202	1.300	0.8476-2.015	0.9852	0.9947	0.5706-1.740
Joint pain	60	13.0	28	17.5	43	19.3	0.1581	0.7036	0.4378-1.158	**0.0308**	0.6248	0.4089-0.9525	0.658	0.8879	0.5217-1.505
Headache	47	10.2	17	10.6	32	14.3	0.8712	0.9527	0.5386-1.682	0.1088	0.676	0.4223-1.086	0.2818	0.7096	0.3812-1.332
Sleep difficulties	56	12.1	18	11.3	27	12.1	0.7693	1.088	0.6206-1.928	0.9959	1.001	0.6236-1.609	0.7971	0.9202	0.4951-1.696
Concentration difficulties	40	8.7	13	8.1	13	5.8	0.8351	1.072	0.5653-2.080	0.1942	1.531	0.8180-2.940	0.3785	1.429	0.6550-3.111
Hair loss	57	12.3	12	7.5	10	4.5	0.0931	1.736	0.9051-3.379	**0.0012**	2.998	1.542-6.247	0.211	1.727	0.6983-4.060

**Reinfection**	**0**		**F**		**B**		**0 vs. F**			**0 vs. B**			**F vs. B**		
	**n**	**%**	**n**	**%**	**n**	**%**	**p**	**OR**	**95% CI**	**p**	**OR**	**95% CI**	**p**	**OR**	**95% CI**
Taste loss	2	4.2	1	2.6	1	2.3	>0.9999	1.652	0.1856-24.50	>0.9999	1.826	0.2053-27.02	>0.9999	1.105	0.05682-21.45
Smell loss	2	4.2	3	7.7	2	4.7	0.6531	0.5217	0.08960-2.685	>0.9999	0.8919	0.1351-5.894	0.6648	1.708	0.3316-9.979
Fatigue	17	35.4	19	48.7	16	37.2	0.2746	0.5772	0.2347-1.368	>0.9999	0.9254	0.389-2.213	0.3724	1.603	0.6516-3.660
Respiratory difficulties	5	10.4	9	23.1	7	16.3	0.1454	0.3876	0.1330-1.321	0.5382	0.598	0.1944-1.961	0.5784	1.543	0.5061-4.299
Joint pain	7	14.6	7	17.9	5	11.6	0.7724	0.7805	0.2647-2.306	0.7629	1.298	0.3967-3.995	0.536	1.663	0.4974-5.196
Headache	6	12.5	6	15.4	8	18.6	0.761	0.7857	0.2146-2.883	0.5625	0.625	0.1879-1.876	0.7746	0.7955	0.2344-2.357
Sleep difficulties	5	10.4	4	10.3	4	9.3	>0.9999	1.017	0.2726-3.518	>0.9999	1.134	0.3057-3.903	>0.9999	1.114	0.3053-4.057
Concentration difficulties	2	4.2	2	5.1	2	4.7	>0.9999	0.8043	0.1219-5.336	>0.9999	0.8913	0.1351-5.894	>0.9999	1.108	1.669-7.338
Hair loss	3	6.3	0	0.0	0	0.0	0.2494	∞	0.7161-∞	0.244	∞	0.7906-∞	>0.9999	/	/

Supplementary Table 4. COVID-19 full vaccination,1st booster vaccination, primary infection, and percental reinfection rates among the respondents and the Czech general population.

	III	IV	V	VI	VII	VIII	IX	X	XI	XII	I	II	III	IV	V	VI	VII	VIII	IX	X	XI	XII	I	II	III	IV	V
	2020										2021												2022				
Full vaccination-dentists											1.5	22.4	43.3	53.6	62.2	69.4	72.3	75.4	77.8	80	82.2	83.6	84.5	85	85.1	85.6	85.8
Full vaccination-general population											0.5	3	6.4	12.4	18.4	40.3	58.2	65.5	67.3	68.4	71.1	73.5	74.4	74.8	74.9	74.9	74.9
1st booster-dentists															0.2	0.2	0.4	0.6	3.2	17.8	42	58.3	64.7	67	68.2	69.3	70.1
1st booster-general population															0	0	0	0	0.1	2.7	10.9	29.5	45	47.5	48.4	49	49.4
Primary infection-dentists	0.5	0.5	0.6	0.6	0.6	0.8	1.2	5.2	6.8	10.4	12.6	14.3	15.4	16.2	16.3	16.3	16.3	16.5	16.8	18.5	22.4	24.8	29	34.7	39.3	41.3	41.7
Primary infection-general population	0	0.1	0.2	0.2	0.3	0.4	0.8	3.8	7	9.5	13.1	16.3	20.3	22.3	22.9	23.1	23.2	23.4	23.6	24.2	27.9	32.2	37.3	44.6	47.9	49.6	49.9
Reinfection-dentists			0.1	0.1	0.1	0.1	0.1	0.1	0.1	0.1	0.1	0.1	0.1	0.2	0.3	0.3	0.3	0.3	0.3	0.5	0.7	0.9	2.8	3.7	5	5.7	6
Reinfection-general population					0	0	0	0	0	0	0	0	0	0.1	0.1	0.1	0.1	0.1	0.1	0.1	0.2	0.3	1	2.3	2.9	3.2	3.3
